# Bile acid signalling and its role in anxiety disorders

**DOI:** 10.3389/fendo.2023.1268865

**Published:** 2023-11-23

**Authors:** Simin Chen, Qi Shao, Jiayi Chen, Xinyi Lv, Jing Ji, Yan Liu, Yuehan Song

**Affiliations:** College of Traditional Chinese Medicine, Beijing University of Chinese Medicine, Beijing, China

**Keywords:** anxiety disorders, bile acid signaling, FXR, TGR5, FGF19, GLP-1

## Abstract

Anxiety disorder is a prevalent neuropsychiatric disorder that afflicts 7.3%~28.0% of the world’s population. Bile acids are synthesized by hepatocytes and modulate metabolism via farnesoid X receptor (FXR), G protein-coupled receptor (TGR5), etc. These effects are not limited to the gastrointestinal tract but also extend to tissues and organs such as the brain, where they regulate emotional centers and nerves. A rise in serum bile acid levels can promote the interaction between central FXR and TGR5 across the blood-brain barrier or activate intestinal FXR and TGR5 to release fibroblast growth factor 19 (FGF19) and glucagon-like peptide-1 (GLP-1), respectively, which in turn, transmit signals to the brain via these indirect pathways. This review aimed to summarize advancements in the metabolism of bile acids and the physiological functions of their receptors in various tissues, with a specific focus on their regulatory roles in brain function. The contribution of bile acids to anxiety via sending signals to the brain via direct or indirect pathways was also discussed. Different bile acid ligands trigger distinct bile acid signaling cascades, producing diverse downstream effects, and these pathways may be involved in anxiety regulation. Future investigations from the perspective of bile acids are anticipated to lead to novel mechanistic insights and potential therapeutic targets for anxiety disorders.

## 1 Introduction

As is well documented, anxiety disorder is a common neuropsychiatric disorder affecting 7.3% to 28.0% of the world’s population (1, 2). Its incidence has increased by 2.3 times in recent years due to the impact of COVID-19 (3), making it the sixth-largest disability disease globally. Of note, it is associated with an increase in the risk of suicidal behavior and cardiovascular disease (4). To date, the pathogenesis of anxiety disorders remains elusive.

However, metabolic disorders, encompassing bile acid (BA) disorders, have recently garnered extensive attention in anxiety research (5). Bile acids are multifunctional endocrine factors that govern lipid and energy metabolism by coordinating the activation of FXR and TGR5 to modulate cellular signaling. Additionally, bile acids also communicate with the central nervous system, traversing the blood-brain barrier (BBB) to bind to their cognate receptors within the brain parenchyma (6).

Emerging evidence from clinical and preclinical studies suggests that bile acid dysregulation may contribute to the development of mental health disorders like anxiety. Earlier clinical investigations have established a direct correlation between bile acid concentration and anxiety symptoms. Moreover, this relationship extends beyond anxiety, as bile acids have been shown to influence anxiety-like behavior in patients with irritable bowel syndrome and other gastrointestinal disorders (7, 8). Earlier studies have also described that bile acids transmit signals to the brain through both direct or indirect pathways and participate in the development of brain diseases, thereby implying a correlation between bile acids and mental illnesses. Elevated serum bile acids have been proven to increase BBB permeability and transmit signals to the central nervous system via FXR, TGR5, etc., eventually elevating the risk of mental disorders (9).However, the underlying mechanisms by which bile acid signals affect the occurrence of anxiety behavior have not been fully elucidated (10). The article is divided into two main sections. The first section provides an overview of the metabolism of bile acids and their different functions and corresponding receptors. The second section consolidates the available evidence on bile acids exerting their effects and transmitting signals through distinct pathways and tissues/organs to influence anxiety, offering a direction for the development of novel treatment strategies from the perspective of bile acids.

## 2 Synthesis, metabolism, and circulation of bile acids

**Figure 1:**
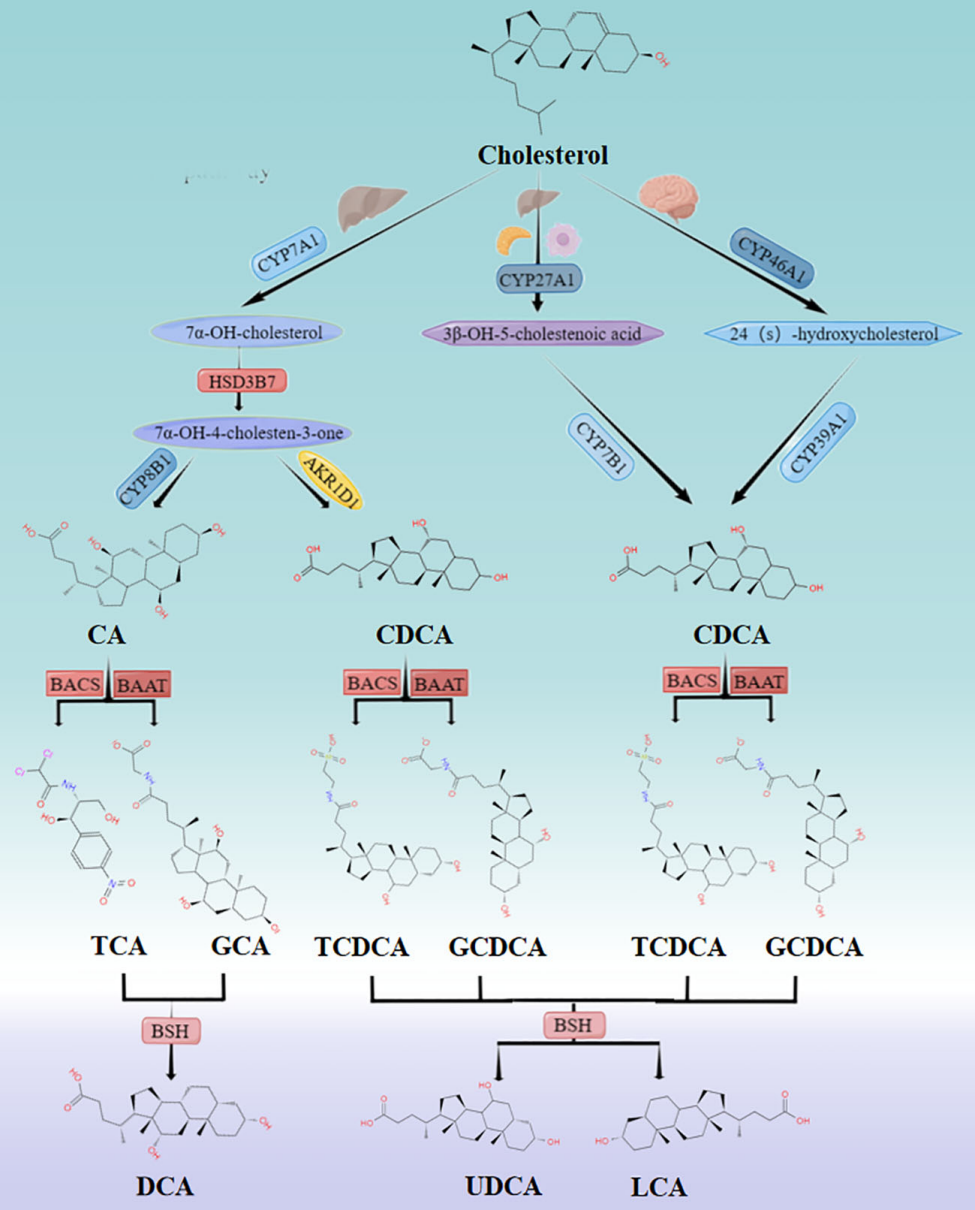
Classic and alternative pathways for bile acid synthesis. Cholesterol undergoes multiple enzymatic reactions to form primary bile acids CA and CDCA. The solubility of these bile acids is increased, and their cell toxicity is reduced by coupling with glycine or taurine. The primary bile acids are then stored in the gallbladder and secreted into the intestine after food consumption. In the intestine, they are further metabolized into secondary bile acids by the intestinal flora. While approximately 95% of bile acids enter the enterohepatic circulation and return to the liver, the remaining 5% are excreted through feces.

Primary bile acids such as cholic acid and chenodeoxycholic acid are synthesized from cholesterol in hepatocytes by two pathways, namely the classic or neutral pathway and the alternative or acidic pathway (11) (**Figure 1**). The former is initiated by cholesterol 7α-hydroxylase (CYP7A1) in the liver, while the latter is triggered by sterol 27-hydroxylase (CYP27A1) in the liver, macrophages and adrenal glands, and cytochrome P450 46A1 (CYP46A1) in the brain (12). In the classic pathway, CYP7A1 catalyzes the conversion of cholesterol to 7α-hydroxycholesterol, which is then converted to 7α-hydroxy-4-cholesten-3-one by 3β-hydroxysteroid dehydrogenase type 7 (HSD3B7). The latter is thereupon converted to cholic acid (CA) by 12α-hydroxylase (CYP8B1) or to chenodeoxycholic acid (CDCA) by Aldo-keto reductase Family 1 Member D1 (AKR1D1). In the alternative pathway, CYP27A1 generates 3β-hydroxy-5-cholenoic acid, which is subsequently converted to CDCA by 7α-hydroxylase (CYP7B1) (11). CDCA is also generated under the action of Cytochrome P450 Family 39 Subfamily A Member 1(CYP39A1) by CYP46A1 (12). Bile acid production is strictly controlled by cytochrome CYP8B1, which determines the amount of cholic acid and chenodeoxycholic acid in the bile acid pool. It is worthwhile emphasizing that in mice, CDCA is converted to ursodeoxycholic acid (UDCA), α-muricholic acid (α-MCA), and β-muricholic acid (β-MCA) by the cytochrome P450 2C70 (CYP2C70) enzyme (13).

Bile acids are conjugated with glycine (primarily in humans) or taurine (primarily in mice) by bile acid-CoA synthase (BACS) and bile acid CoA:amino acid N-acyltransferase (BAAT) before secretion in the liver increases solubility and reduce cellular toxicity (12). This process involves the conjugation of CA to generate glycocholic acid (GCA) or taurocholic acid (TCA) and the coupling of CDCA to yield glycochenodeoxycholic acid (GCDCA) or taurochenodeoxycholic acid (TCDCA). In mice, coupling generates tauro-α-muricholic acid (TαMCA), tauro-β-muricholic acid (TβMCA), and tauro-ursodeoxycholic acid (TUDCA), which are then transported and stored in the gallbladder by the bile salt export pump (BSEP) (12).

Following food intake, the duodenum secretes cholecystokinin (CCK), which promotes gallbladder contraction and the release of bile acids into the intestine. Bile salt hydrolase (BSH) secreted by specific intestinal bacteria oxidizes and epimerizes specific hydroxyl groups on bile acids, affecting their physicochemical properties and biological toxicity, and converts primary bile acids to secondary bile acids, including deoxycholic acid (DCA), UDCA, and lithocholic acid (LCA), which promote the metabolism of fat and fat-soluble vitamins (14). Approximately 95% of bile acid molecules remain unbound and proceed to the distal end of the ileum, whereby they are reabsorbed by the ileal epithelium via the apical sodium-dependent transporter (ASBT). The reabsorbed BAs are transported through intestinal epithelial cells to organic solute transporter alpha/beta (OSTα/β) on the sinusoidal membrane and are transported back to the liver via sodium (Na+)-taurocholate co-transporting polypeptide (NTCP) and organic anion transporters (OATP) through the portal vein to complete the enterohepatic circulation. Noteworthily, a small proportion of BAs (up to 10%) escape into the systemic circulation without undergoing hepatic reabsorption (15) and reach the brain by simple diffusion (16) or active transport (17) across the BBB.

Bile acid synthesis and enterohepatic circulation primarily occur in the liver, gallbladder, intestine, and brain. Evidence of bile acid production in the brain has also emerged. In addition to those taken up from systemic circulation, BAs synthesized by the brain have been detected in rodent and human brains. For instance, CA, CDCA, and deoxycholic acid (DCA) were detected in rat brain tissue cytoplasm (18), whereas other bile acids were identified in the cortex of Alzheimer’s disease patients, including glycocholic acid (GCA), LCA, and UDCA, among others (19). However, the pathological and physiological processes involved in brain-synthesized bile acids are currently unclear, making it difficult to describe their function.

## 3 Various bile acids and corresponding receptors are involved in systemic metabolism and immune regulation

The metabolism of bile acids is contingent upon the interaction between bile acids and their receptors, which are expressed in the liver, intestine, brain, etc. Nuclear receptors include FXR, pregnane X receptor (PXR), vitamin D receptor (VDR), and constitutive androstane receptor (CAR), while membrane receptors include TGR5 and sphingosine-1-phosphate receptor 2 (S1PR2) (20). They strictly govern the synthesis, absorption, and excretion of bile acids. Variations in the source and structure of bile acids impart unique physicochemical properties and functional characteristics that naturally translate into diverse effects on their corresponding receptors. To explore these differences, the functions of various bile acids were detailed based on their receptor targets, as illustrated in **Table 1**.

**Table 1:** Functional involvement of different bile acids and their corresponding receptors.

Bile Acid Ligands	Receptor	Cellular Localization	Main Function	References
CDCA, DCA, LCA, CA	FXR	Intestine	Controlling bile acid synthesis by inhibiting CYP7A1 via FGFs/FGFRs	(21)
CDCA, DCA, LCA, CA		Liver	Suppressing CYP7A1 and CYP8B1 via SHP-induced inhibition of bile acid synthesis	(22)
CA, TCA			Regulation of lipid metabolism by SHP inhibition of SREBP-1c	(23)
CDCA, DCA		Hippocampus	Promoting depression by inhibiting BDNF/TrkB	(24)
CDCA			Regulation of glucose metabolism by enhancing insulin sensitivity in the brain	(25)
CDCA		Prefrontal cortex	Relieving depression through the NLRP3/GluA1 signaling pathway	(26, 27)
CA	TGR5	Kupffer cells	Mediating the inflammatory response through JNK-dependent pathways	(28)
Taurine		Sinusoidal Endothelial Cell	Preventing oxidative stress by inducing ENOS	(29)
HCA		Intestine	Stimulating the secretion of GLP-1 by intestinal L cells and regulating glucose metabolism	(30, 31)
TUDCA		Neuron	Regulating Sirtuin3 signaling to protect against apoptosis	(32)
TUDCA		Microglia	Upregulating cAMP expression and inhibiting microglial activation	(33)
TCDCA		Astrocyte	Through AKT/NF κ B signaling pathway inhibits neuroinflammation	(34)
TLCA		Ventricle	Increase fat oxidation and regulate lipid metabolism	(35)
DCA, TDCA, TCA		Hypothalamus	Activating SNS to promote energy metabolism and reduce fat content	(36)
LCA	PXR, VDR	Liver	Alleviating liver toxicity of LCA through enzymes that regulate bile acid metabolism	(37)
	CAR, PXR, VDR	Intestine	Maintaining bile acid homeostasis by inhibiting CYP7A1 through FGF15/FGF19	(38)
TCA, GCA, GDCA, TDCA, TUDCA	S1PR2	Liver	Promoting liver inflammation through ERK1/2/NF- κ B/COX-2	(39)
TCA		Neuron	Upregulating CCL2 expression and aggravating neuroinflammation	(40)

### 3.1 FXR

FXR is a ligand-activated transcription factor that plays a crucial role in regulating bile acid homeostasis and participates in enterohepatic circulation. FXR can be activated by several bile acids, with hydrophobic bile acid CDCA being the most effective ligand for FXR. The order of affinity of bile acids to FXR is as follows: CDCA > DCA > LCA > CA (41). FXR is largely expressed in the liver, intestine, kidney, adrenal gland, etc. (42), as well as in cortical neurons of the brain (43, 44).

Intestinal FXR is a crucial regulatory factor that maintains physiological enterohepatic circulation. Indeed, FXR acts as a sensor for elevations in bile acid levels (especially CDCA). Specifically, intestinal FXR stimulates fibroblast growth factor 15 (FGF15) in mice or FGF19 in humans located at the distal portion of the ileum. After crossing the portal vein, it activates the liver fibroblast growth factor receptor (FGFR) to inhibit cholesterol 7α-hydroxylase (CYP7A1) and mediate bile acid synthesis in liver cells (21). This is the primary pathway of bile acid negative feedback. Interestingly, FGF15/19 has been found to enter the systemic circulation and cross the blood-brain barrier to reach the brain, where it binds with FGFR4 in the brain and regulates brain function (6). Meanwhile, FGFR4 has been detected in the hypothalamus and cholinergic neurons located in the nucleus accumbens (45). The bile acid-mediated FXR-FGF15/19 pathway not only establishes a connection between the liver and intestine but also extends to various brain regions.

Hepatic FXR is a key molecule for synthesizing bile acids. Under physiological conditions, activation of FXR by the CDCA and CA primary bile acids plays a decisive role in maintaining hepatic bile acid homeostasis. This process involves hepatic FXR inhibition of 7-ketocholesterol synthesis through the small heterodimer partner (SHP) α Hydroxylase enzyme, resulting in the suppression of bile acid synthesis and prevention of hepatic bile acid accumulation (22). This negative feedback loop involving bile acids and FXR represents the second bile acid regulatory pathway. CA and TCA have also been determined to play key roles in the regulation of lipid metabolism. In other words, these bile acids activate the FXR-SHP pathway to inhibit Sterol Regulatory Element Binding Protein-1c (SREBP-1c), a transcription factor that controls hepatic lipid biosynthesis (23).

FXR expression has been detected in cortical neurons of both humans and mice. *In vitro* cultured neurons express FXR in their nuclei, whereas *in vivo* neurons express FXR in the cytoplasm (43). Moreover, FXR is present in various brain tissues, such as the hippocampus, cerebellum, and frontal cortex. Experimental data indicate that CDCA and DCA may downregulate hippocampal FXR expression, which in turn increases the level of brain-derived neurotrophic factor (BDNF), thereby exerting anti-depressant effects (24). However, CDCA exerts anti-depressant actions in the mouse prefrontal cortex by upregulating FXR expression to inhibit NOD-like receptor protein 3 (NLRP3) inflammasome activation and increase GluA1 levels (26, 27), highlighting the diverse regional effects of bile acid-FXR interactions in the brain. Additionally, CDCA binding to FXR in the hippocampus of AlCl3-treated rats can also enhance insulin sensitivity (25).

### 3.2 TGR5

TGR5 is a G protein-coupled receptor implicated in bile acid metabolism (6)and is activated by hydrophobic bile acid LCA, which has the highest affinity for TGR5 among other bile acids such as DCA, CDCA, and CA (6). TGR5 is abundantly expressed in various tissues and cells, including the intestine, gallbladder, liver sinusoidal endothelial cells (SEC), etc. (46), and its expression in the brain has also been gradually recognized (47, 48). It affects glucose and energy metabolism and plays a role in immune regulation.

Activation of TGR5 signaling in Kupffer cells and SEC has been noted to promote anti-inflammatory responses. Specifically, CA principally plays a pro-inflammatory role in Kupffer cells, driving the production of pro-inflammatory cytokines in the liver through the TGR5/c-Jun N-terminal kinase (JNK)-dependent pathway (28). As a protective bile acid, taurine is more likely to bind to TGR5 in SECs to stimulate endothelial nitric oxide synthase (ENOS) and prevent oxidative stress-mediated inflammatory reactions (29).

TGR5 has a wide range of functions in the intestine. Hyocholic acid (HCA) activates TGR5, triggering the secretion of GLP-1 from intestinal L cells to regulate glucose metabolism (30). Notably, HCA has been proposed as a biomarker for glucose metabolism disorders in clinical trials (31). Additionally, only a quarter of GLP-1 enters the portal vein of the liver. Among them, 10-15% enter the body circulation to activate GLP-1 receptors (GLP-1R) located in the terminal bed nucleus, hippocampus, and paraventricular nucleus of the hypothalamus (6) or transmit signals to CNS through the vagus nerve-brainstem-hypothalamus pathway (6).

TGR5 in the brain is chiefly expressed in cortical neurons, astrocytes, and microglia. Unlike brain FXR, the activation of TGR5 exerts a positive effect on brain function. Known for its neuroprotective effects, TUDCA has been shown to mitigate cell apoptosis through activation of the TGR5/Sirtuin3 signaling axis in neurons (32). Besides, the neuroprotective actions of TUDCA extend beyond its impact on neurons, as it has also been documented to regulate neuroinflammation by modulating the TGR5/cAMP pathway and prevent microglial activation (33). Similarly, taurocholic acid deoxycholic acid (TCDCA), another conjugated bile acid, has been observed to possess anti-inflammatory and immune regulatory properties that mainly operate within astrocytes. Its actions are mechanistically linked to the TGR5/threonine kinase (AKT)/nuclear factor κB (NF-κB) signaling axis and suppressing neuroinflammation (34). Comparable to the CNS FXR receptor, TGR5 within the CNS also regulates lipid and energy metabolism. The administration of taurolithocholate (TLCA), the most potent natural TGR5 agonist, through intracerebroventricular infusion in mice has been found to increase fat oxidation and limit fat mass (35). Additionally, three bile acids, namely DCA, taurine deoxycholic acid (TDCA), and TCA, target hypothalamic TGR5 to promote energy metabolism through the sympathetic nervous system (SNS) to reduce fat content (36).

### 3.3 PXR, CAR, and VDR

Although there is no specific bile acid ligand that binds with CAR, PXR, CAR, and VDR can all promote the clearance of hepatotoxic LCA. Accumulation of LCA leads to activation of PXR and VDR, and CAR acts as an indirect sensor of bile acids to transcriptionally regulate the expression of bile acid-related enzymes and transporters (37). In the intestines, PXR, CAR, and VDR inhibition of CYP7A1 action occur through the FGF15/FGF19 pathway to maintain BA homeostasis (38).

### 3.4 S1PR2

S1PR2 has been essentially implicated in promoting inflammatory responses, with heightened expression levels observed in the liver. S1PR2 activation by ligands such as TCA, GCA, Glycodeoxycholic acid (GDCA), TDCA, and TUDCA leads to membrane trafficking and activation of ERK1/2 kinases, which results in the nuclear translocation of NF-κB transcription factors. This nuclear translocation then drives the transcriptional upregulation of cyclooxygenase-2 (COX-2), an enzyme involved in hepatic inflammation (39). In addition to its hepatic expression, S1PR2 is also abundantly expressed in neurons within the CNS. Circulating TCA has been theorized to penetrate the CNS and bind S1PR2 on neurons, potentiating chemokine ligand 2 (CCL2)-induced neuroinflammation and microglia activation, resulting in hepatic encephalopathy that is exacerbated by hepatic dysfunction (40).

## 4 Signal transduction of bile acids and their receptors in patients with anxiety disorders

Clinical studies have reported that in patients with anxiety disorders, certain metabolically abnormal bile acids excessively activate FXR and promote the secretion of FGF19 in intestinal cells. The latter enters the brain through systemic circulation and participates in the neuroinflammatory process, thereby promoting anxiety (49). A large number of studies have consistently demonstrated that anxiety related to abnormal bile acid metabolism is frequently accompanied by intestinal diseases (7, 8, 10, 50), particularly irritable bowel syndrome (IBS). Therefore, it is essential to emphasize that changes in bile acids are closely related to anxiety disorders in IBS patients. Excessive CA and DCA affect the expression of TGR5 in colonic mucosa, activate the TGR5-JNK pathway of intestinal epithelial cells, and cause an abnormal number and distribution of cell connections, leading to damage to the intestinal mucosal barrier and increased permeability. On the other hand, activating the TGR5/5-hydroxytryptamine (5-HT) signaling axis of intestinal enterochromaffin cells conduces to visceral hypersensitivity, which is particularly relevant to the occurrence of IBS (51, 52). These effects of intestinal mucosal barrier impairment and visceral hypersensitivity due to the influence of the gut-brain axis ascend along the afferent nerves in the intestine to the central nervous system, affecting the release of 5-HT and γ-aminobutyric acid, thereby exacerbating anxiety in IBS patients. Indeed, compelling evidence suggests that bile acids affect anxiety through different pathways (53). As previously mentioned, FXR and TGR5 are the most extensively researched and distinctive receptors of bile acids. These two receptors are widely expressed and play a crucial role in the liver-gut axis and brain. In light of the discussions in Sections 3.1 and 3.2, this article exclusively focused on anxiety and offered a more comprehensive explanation of the mechanisms by which bile acids and their receptor signaling pathways influence anxiety.

### 4.1 Bile acids transmit signals that affect anxiety via the direct pathways

**Figure 2:**
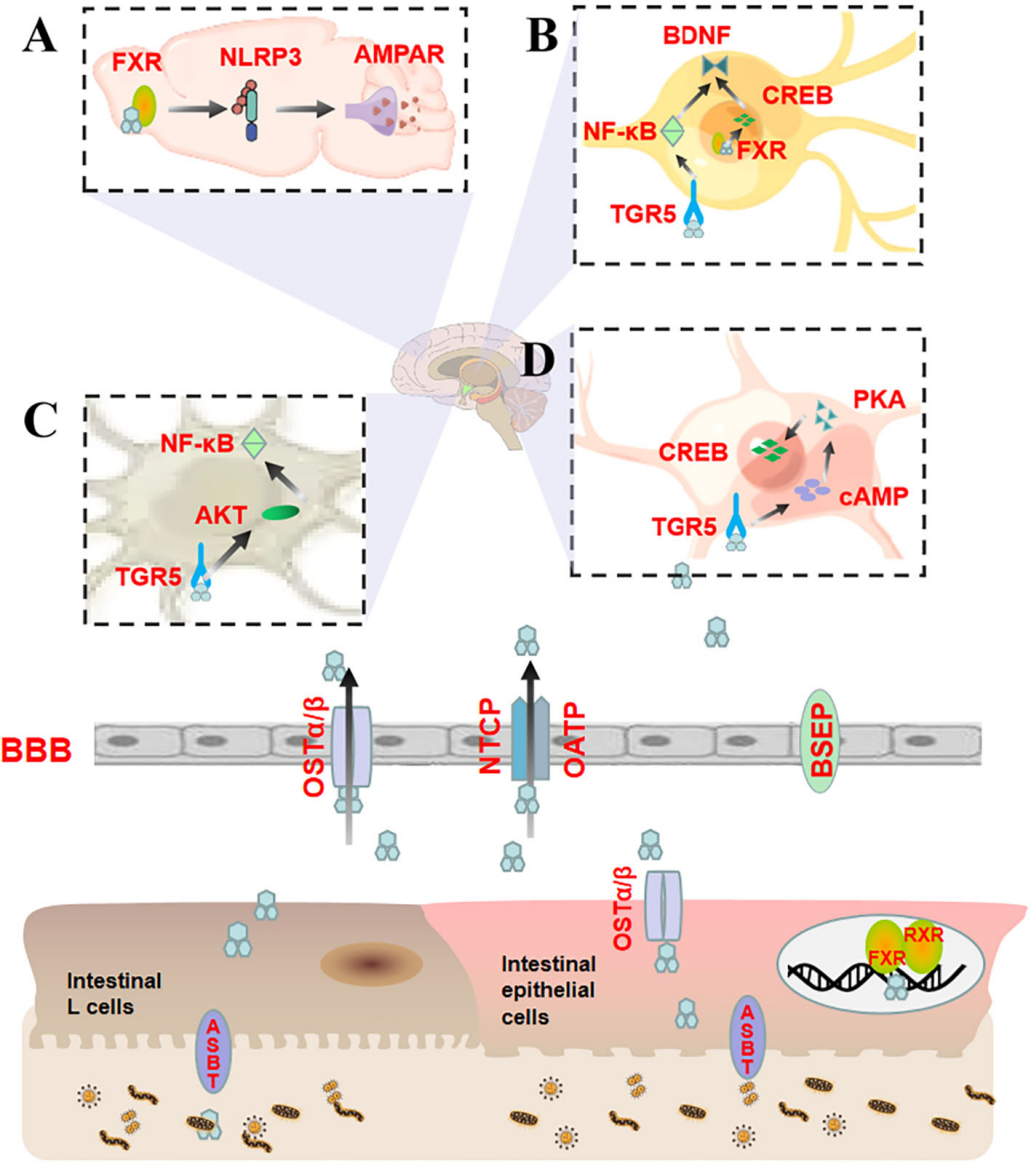
Bile acids entering different regions of the brain directly regulate anxiety by binding to FXR and TGR5. **(A)** CA activates FXR in the prefrontal cortex, which inhibits the expression of NLRP3 inflammasomes and restores AMPAR levels, thereby affecting LTP and synaptic plasticity; **(B)** CDCA activates FXR on hippocampal neurons and targets CREB and BDNF through a CREB-dependent mechanism, reducing neural function. TUDCA activates TGR5 on hippocampal neurons to relieve neuroinflammation by lowering NF-κB and increasing BDNF levels, thus exerting neuroprotective effects; **(C)** TUDCA activates TGR5 in astrocytes in the cerebral cortex, upregulates AKT expression, and decreases NF-κB levels; **(D)** INT-777 activates TGR5/cAMP/PKA on microglia to counteract neuroinflammation and promote CREB phosphorylation.

According to earlier studies, unconjugated bile acids such as CA, DCA, and CDCA (54) can diffuse across the BBB in a concentration-dependent manner. At the same time, experimental studies have identified a positive correlation between the levels of CA, CDCA, and DCA in the brain and their serum levels (18). At high concentrations (≥1.5 mM), bile acids can function as detergents to dissolve endothelial cell membranes and thus disrupt the lipid layer of the BBB (16). At low concentrations (0.2-1.5 mM), bile acids enhance BBB permeability by a rac1-dependent phosphorylation mechanism related to tight junction-associated proteins (55)and diffuse into the brain. Conjugated bile acids require active transport across the BBB with the help of transport proteins such as NTCP, OATP, OSTα/β, and BSEP in the blood-brain barrier and choroid plexus. This is ascribed to the presence of both hydrophilic hydroxyl and carboxyl groups and hydrophobic methyl groups (17, 56, 57). BAs interact with FXR and TGR5 in the brain through the aforementioned mechanisms. Although the primary objective of this process is to transport excess cholesterol from the brain to the circulatory system (58), BAs also affect brain functions such as emotions (58). The regulation of emotions is fundamentally dependent on different parts of the brain cortex, such as the prefrontal cortex and hippocampus. Of note, different bile acid ligands and receptors have varying effects when acting on different parts (**Table 2** and **Figure 2**).

**Table 2:** Different bile acids transmitting signals to affect anxiety.

Bile Acid Ligands	Cellular Localization	Pathways	Mechanisms	References
CDCA	Hippocampal neurons	FXR/CREB/BDNF	CREB dependency	(25, 59, 60)
CA	Prefrontal cortex	FXR/NLRP3/AMPARs	Synaptic plasticity	(27, 61, 62)
INT-777	Microglia	TGR5/cAMP/PKA/CREB	Microglia activation	(63–66)
TUDCA	Hippocampal neurons	TGR5/NF-κB/BDNF	Neuroprotection	(67–71)
	Astrocyte	TGR5/AKT/NFκB	Neuroinflammation	(34, 72–75)

CDCA activates FXR in hippocampal neurons after diffusing through the BBB. This activation inhibits the activity of the cAMP response element-binding protein (CREB) and the expression of BDNF (25). The former is a transcription factor that regulates gene transcription and is an important transcription element necessary for long-term memory and neuronal survival. Furthermore, it can regulate the transcription and expression of BDNF and induce anxiety-like behavior. Contrastingly, the latter is a protein with neurotrophic effects that can enhance learning and memory abilities. It is also a downstream molecule of CREB and affects anxiety and other mental illnesses. Studies have evinced that CREB and BDNF expression levels are down-regulated in the hippocampus of rats with anxiety-like behavior (59, 60), suggesting that the overactivation of FXR in hippocampal neurons by excessive CDCA may target and inhibit the CREB/BDNF pathway via a CREB-dependent mechanism to impair neural function and promote anxiety.

CA, a natural ligand of FXR (76), can activate FXR in the prefrontal cortex (PFC) and suppress the expression of NLRP3 inflammasome, thereby restoring the level of α-amino-3-hydroxy-5-methyl-4-isoxazole-propionic acid receptors (AMPARs) (27). NLRP3 is an inflammasome sensor protein that aggravates anxiety-like behavior by inducing the activation of microglia in PFC (61, 62). It is noteworthy that NLRP3 also participates in long-term potentiation (LTP) mediated by AMPARs (77), which are tetramers composed of four homologous core subunits. The dynamic expression of AMPARs in the postsynaptic membrane is associated with LTP. Their synaptic transmission efficiency is a crucial factor in the LTP process that is responsible for synaptic plasticity. The weakening of this efficiency can impair LTP (78), reducing synaptic activity and quantity, especially in the PFC, which can cause anxiety (79, 80). It is evident that CA activates the prefrontal cortex FXR and dynamically balances anxiety via the NLRP3 inflammasome/AMPARs signaling pathway.

INT-777 is a modified bile acid analog that acts as a specific TGR5 agonist. The activation of the TGR5-cAMP-protein kinase A (PKA) axis exerts an anti-inflammatory effect on microglia, which in turn promotes the phosphorylation of the target protein CREB. The cAMP/PKA/CREB signaling pathway is closely associated with anxiety (63–66). The anti-anxiety effect of INT-777 is elicited through the activation of microglial cells, which modulate the TGR5/cAMP/PKA/CREB axis.

TUDCA is an agonist of TGR5 and confers neuroprotective effects on the central nervous system. It activates TGR5 in hippocampal neurons to mediate NF-κB/BDNF signaling and mitigate neuronal apoptosis (67). NF-κB is a transcription factor that swiftly responds to harmful stimuli and is highly expressed in inflammatory reactions (68). Its expression is negatively correlated with BDNF expression in hippocampal neurons and impairs neuronal development and synaptic plasticity (69–71), resulting in anxiety-like behavior.

TUDCA is known to activate the TGR5/AKT/NF-κB signaling pathway in astrocytes in the cerebral cortex (34). AKT, also referred to as protein kinase B, is a widely expressed protein in the cerebral cortex with a molecular weight of approximately 60 kDa (72). Its upregulation in astrocytes is associated with anxiety-like behavior in mice (73). Moreover, it can lead to NF-κB phosphorylation and participate in the occurrence and development of anxiety disorders (74, 75). TUDCA regulates the TGR5/AKT/NFκB signaling pathway through astrocyte-mediated inflammatory processes, thus playing a role in anxiety.

### 4.2 Bile acids transmit signals affecting anxiety via indirect pathways

**Figure 3:**
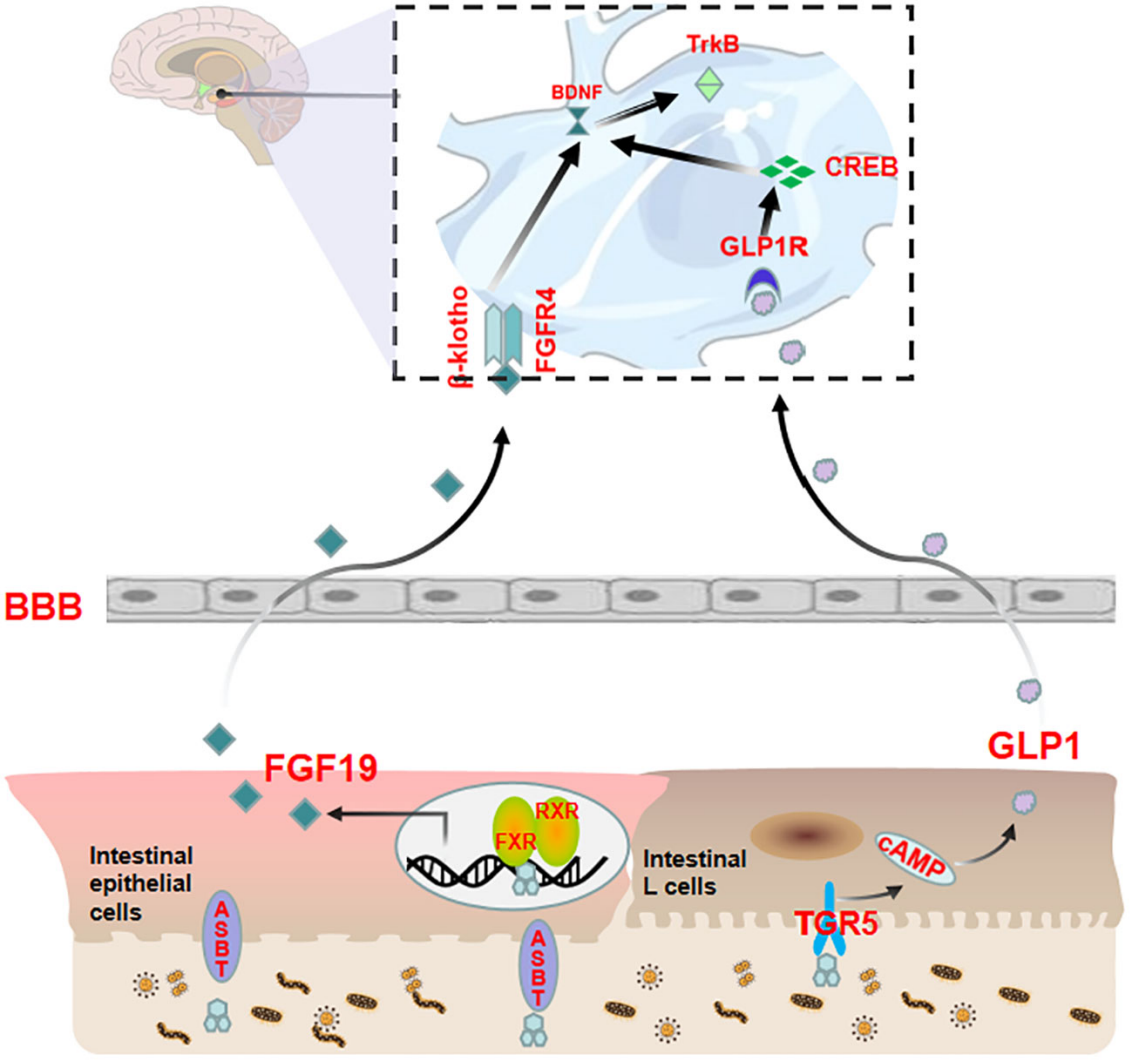
Bile acids modulate anxiety by indirectly transmitting signals to the brain via the FXR-FGF19 and TGR5-GLP-1 pathways upon entering the intestine. Bile acids enter the intestine and activate FXR in ileal enterocytes to upregulate FGF19 expression. In addition to returning to the liver through the portal vein, a portion of FGF19 crosses the BBB to interact with FGFRs in the brain and affect the BDNF-TrkB signaling pathway. Bile acids can also stimulate intestinal L cells to release GLP-1. A proportion of GLP-1 reaches the portal vein, and a smaller amount reaches the brain to interact with GLP-1R, thereby affecting the CREB/BDNF signaling pathway.

In addition to direct pathways, bile acids can also affect anxiety via indirect pathways. As aforestated, bile acids initiate the FXR-FGF19 and TGR5-GLP-1 pathways following their release into the intestine to transmit signals to the central nervous system. This section aimed to focus on the mechanisms by which these two pathways affect anxiety in the brain (**Figure 3**).

Activation of FXR by fasting serum CDCA has been shown to independently modulate FGF19 in clinical studies (81, 82). FGF19 is a member of the FGF family that is predominantly expressed in the intestinal epithelial cells located at the terminal ileum (83, 84). FGFs are widely distributed throughout the central nervous system and are known to play a central role in neuronal function, development, and metabolism. Recent studies have revealed that FGFs have a significant impact on the occurrence and development of mental illnesses such as anxiety disorders, depression, schizophrenia, bipolar affective disorder, etc., and are anticipated to serve as novel biomarkers for the diagnosis and prognosis of mental illnesses (85, 86). The intestinal FXR-FGF19 complex partially diffuses into the hepatic portal vein and binds with the auxiliary receptor β-Klotho in the liver to activate FGFRs, thereby mediating the negative feedback pathway of bile acids. The remaining part of the complex subsequently circulates throughout the body and binds with FGFRs of the brain. Among the four types of FGFRs, FGFR4 is mainly distributed in the hypothalamus (87) and cholinergic neurons (45) in the medial habenula, with a lower proportion of β-Klotho receptors (88). FGFR4 mainly participates in emotional regulation (89). An unbalanced FGFs/FGFRs system can cause glucose metabolism disorders, neural inflammation, hypothalamic–pituitary–adrenal axis hyperfunction, BBB damage, neuroplasticity reduction, neuronal apoptosis, etc., which affect the structure and function of the cerebral cortex, hippocampus, hypothalamus, pituitary and other tissues, ultimately resulting in emotional disorder (89). Among them, aberrant glucose metabolism is closely related to anxiety; that is, insulin resistance may play a pivotal role in the development of emotional disorders (90–92). Similarly, HPA axis dysfunction caused by endocrine disorders is an inducing or aggravating factor for anxiety (93–95). Recent studies have shown that regulating FGFR can affect BDNF expression (96) and that the BDNF-TrkB signaling pathway plays an important role in anxiety (96, 97). The overexpression of serum CDCA over-activates intestinal FXR receptors, resulting in an imbalance in the FGFs/FGFRs system that impacts the BDNF-TrkB signal pathway, which finally culminates in anxiety-like behavior.

Several studies have indicated that bile acid can stimulate intestinal TGR5 to release GLP-1, with LCA being the most potent agent in this process (98–101). GLP-1 is a type of enterotropic insulin produced by specific intestinal endocrine cells (L cells) and secreted in large quantities after food intake. It can regulate glucose uptake and insulin resistance in the brain, thereby improving neuroinflammation and neurogenesis (102). Additionally, GLP-1 plays a crucial role in synaptic plasticity and emotion regulation (103) and may be a potential key regulator of anxiety behavior (104–106). The indirect TGR5-GLP-1 signaling pathway can interact with GLP-1R in the brain through systemic circulation to affect central nervous system function (6). This interaction can concurrently stimulate CREB/BDNF to modify brain synaptic plasticity and participate in anxiety regulation (106). Alternatively, it can regulate anxiety emotions through the vagus nerve-brainstem-hypothalamus pathway. The vagus nerve is a regulator of mental illness (107–109), and its incoming fibers stimulate the monoaminergic brain system in the brainstem (107), which then plays a key role in anxiety emotions in the hypothalamus (110).

## 5 Conclusion and perspectives

Anxiety may be associated with bile acids and their signaling pathways. Different bile acids and their corresponding receptors participate in systemic activities, including metabolic and immune regulation and radiating to tissues and organs such as the brain, liver, and intestines. Recent years have witnessed a growing body of research into the effect of bile acids on brain function, providing valuable insights and opening up new avenues of investigation. Bile acids can directly bind to bile acid receptors in the brain through the blood-brain barrier to induce anxiety-like behavior. They can also form receptor-hormone complexes through the enterohepatic circulation to enter the systemic circulation and bind to corresponding hormone receptors in the brain, inducing anxiety. The mechanism of anxiety disorder is complex and is regulated at various levels besides brain lesions. Targeting bile acids may be an efficient approach for exploring the pathological mechanism underlying anxiety disorders. Despite only specific bile acid signaling pathways being related to anxiety, this approach can broaden our understanding of anxiety for the development of new treatment strategies.

## Author contributions

SC: Writing – original draft. QS: Writing – review & editing. JC:Writing – review & editing. XL: Writing – review & editing. JJ: Writing – review & editing. YL: Supervision, Writing – review & editing. YS: Supervision, Writing – review & editing.
